# Impact of Malocclusion, Tooth Loss and Oral Hygiene Habits on Quality of Life in Orthodontic Patients: A Cross-Sectional Study

**DOI:** 10.3390/ijerph18137145

**Published:** 2021-07-03

**Authors:** Dinis Pereira, Vanessa Machado, João Botelho, Luís Proença, João Rua, Carolina Lemos, José João Mendes, Ana Sintra Delgado

**Affiliations:** 1 Orthodontics Department, Clinical Research Unit (CRU), Centro de Investigação Interdisciplinar Egas Moniz (CiiEM), Egas Moniz—Cooperativa de Ensino Superior, 2829-511 Caparica, Portugal; vmachado@egasmoniz.edu.pt (V.M.); anasintradelgado@gmail.com (A.S.D.); 2 Clinical Research Unit (CRU), CiiEM, Egas Moniz—Cooperativa de Ensino Superior, 2829-511 Caparica, Portugal; jbotelho@egasmoniz.edu.pt (J.B.); jrua@egasmoniz.edu.pt (J.R.); jmendes@egasmoniz.edu.pt (J.J.M.); 3 Quantitative Methods for Health Research Unit (MQIS), CiiEM, Egas Moniz—Cooperativa de Ensino Superior, 2829-511 Caparica, Portugal; lproenca@egasmoniz.edu.pt; 4 Population Studies Department, Institute of Biomedical Sciences Abel Salazar (ICBAS), 4050-313 Porto, Portugal; clclemos@ibmc.up.pt; 5 UniGENe, Institute for Molecular and Cell Biology (IBMC), 4050-313 Porto, Portugal; 6 Instituto de Investigação e Inovação em Saúde (i3S), University of Porto, 4050-313 Porto, Portugal

**Keywords:** oral health-related quality of life, malocclusion, orthodontic treatment, age, tooth loss

## Abstract

We aimed to assess the impact of malocclusion on oral health-related quality of life (OHRQoL) in a sample composed of adolescents, younger adults and adults seeking orthodontic treatment. Participants were consecutively enrolled from January 2019 to March 2020. The oral health impact profile (OHIP-14) was used to measure the OHRQoL. The index of complexity, outcome and need (ICON) was used to assess malocclusion. Sociodemographic, medical, and clinical questionnaires were recorded. Statistical analyses were performed according as a function of sex and age range (15–30 or >30 years old). Linear and logistic regression models were applied to assess the association between OHIP-14 total score, malocclusion, and other relevant confounding variables. In a final sample of 93 participants (60 females and 33 males, aged 15 to 60 years), men reported significantly better OHRQoL (*p* = 0.005). Participants aged 30 years or older reported significantly worse OHRQoL (*p* = 0.042). OHIP-14 was significantly correlated with age (ρ = 0.259, *p* < 0.05) and the number of missing teeth (ρ = 0.369, *p* < 0.001). Multivariable regression showed OHIP-14 being associated with the number of missing teeth (B = 1.48, SE = 0.57, *p* < 0.05) and the presence of missing teeth (B = 1.38, SE = 0.65, *p* < 0.05). Malocclusion showed no association with OHRQoL. Age and the number of missing teeth may be key factors on self-perceived OHRQoL in adult patients seeking orthodontic treatment.

## 1. Introduction

Oral health-related quality of life (OHRQoL) is a multidimensional construct of the individual’s subjective assessment on oral health, functional and emotional well-being, expectations and satisfaction [1,2]. Moreover, OHRQoL is an integral part of general health and well-being [2], with an increasing focus on dental research [3]. The assessment of OHRQoL has been made through the development and validation of questionnaires, and the oral health impact profile (OHIP) is a fine example with good psychometric properties [4]. Later, the OHIP was shortened to a more practical tool of 14 questions, the OHIP-14 [5], being considered a more practical instrument in clinical practice and epidemiological surveys, and with good reliability and validity [1].

Malocclusion is defined as an abnormal craniofacial growth and development. Notwithstanding, orthodontics corrects some of the cases, but not all [6,7,8]. Interestingly, research on the association between malocclusion and OHRQoL has been growing, suggesting that malocclusion can influence physical, social and psychological characteristics, and may play a role in social acceptance and interactions [9,10].

Most evidence on the association between malocclusion and OHRQoL has focused on children and adolescents [7,11,12,13]; however, studies involving adults largely focused on participants aged 18 to 25 years [14,15]. In fact, adults are increasingly seeking orthodontic care [16] and the lack of information regarding OHRQoL in adults with malocclusions makes it important to investigate this association in samples with wider adult age ranges. Moreover, the link between malocclusion and OHRQoL in different stages of adulthood has never been explored, even more so in high-income countries where emerging adulthood has extended to about 29 years of age [17].

Therefore, we aimed to investigate the association of malocclusion with OHRQoL in a sample of adolescents, younger adults and adults seeking orthodontic treatment. As secondary outcomes, we investigated whether sociodemographic factors, the number of missing teeth, oral health behaviors, systemic conditions and chronic medications might be confounding variables in this association.

## 2. Materials and Methods

This investigation was approved by the Egas Moniz Ethics Committee (ethical approval no. 769) and was carried out in accordance with the Helsinki Declaration of 1975, as revised in 2013. This study was performed under the Strengthening the Reporting of Observational Studies in Epidemiology (STROBE) guidelines [18] (Table S1).

### 2.1. Study Design

This cross-sectional study consecutively enrolled patients seeking orthodontic treatment, for the first time, at the Orthodontic Department of Egas Moniz Dental Clinic (Almada, Portugal) over a 15-month period, from January 2019 to March 2020. Data collection occurred during the first orthodontic diagnosis appointment, after a detailed explanation with direct contact with the participant (in the case of underage patients, in the presence of the legal guardian) and after obtaining the respective signed informed consent. All data were registered on a database specifically created for this purpose, where a coded number was attributed to each participant to secure confidentiality.

### 2.2. Participants and Eligibility Criteria

As OHIP-14 is recommended to patients of at least 15 years of age [19]; the exclusion criteria were as follows: age below 15 years old; past history or ongoing orthodontic treatment; cleft lip and/or palate; hemifacial microsomia, maxillofacial deformities (due to trauma or tumors); untreated dental caries; the presence of a deep periodontal pocket (periodontal pocket depth ≥4 mm); unable to participate in the survey. Data were collected through both face-to-face interviews and clinical examinations.

### 2.3. Sociodemographic and Medical Questionnaire

During the initial appointment, participants provided sociodemographic information by a self-reported questionnaire. The questionnaire covered questions on the following items: age, sex, educational level, occupation status (student, employed, unemployed or retired), and marital status (single, married/union of fact, divorced or widowed). The educational levels were categorized according to the 2011 International Standard Classification of Education (ISCED-2011) [20] as ‘no education’ (ISCED 0 level), ‘elementary’ (ISCED 1–2 levels), ‘middle’ (ISCED 3–4 levels), or ‘higher’ (ISCED 5–8 levels). In the medical questionnaire, participants reported the presence of systemic diseases (i.e., hypertension, diabetes mellitus, among others) and chronic medications.

### 2.4. Clinical Questionnaire and Examination

The participants’ oral health hygiene habits included the daily frequency of toothbrushing (as a continuous measure: one time, two times or three times per day), dental flossing (categorized as yes or no), and the type of toothbrush (categorized as manual or electric).

The clinical orthodontic parameters were recorded including panoramic x-ray, bite registrations, and upper and lower arch alginate impression (Ruthinium Alginate, Badia Polesine, Italy) and then were casted with dental stone (Pro-Solid Super, Courbevoie, France). 

The number of missing teeth was recorded (as a continuous variable) via clinical observation, assisted by panoramic x-ray and excluding third molars. Additionally, we categorized the missing tooth according to the tooth type: anterior teeth (incisors and canines), premolars, and molars.

Malocclusion was assessed through the index of complexity, outcome and need (ICON) [21]. The ICON is used to evaluate treatment need, treatment outcome and complexity [21], and its aesthetic score relies on the index of orthodontic treatment need (IOTN) [22]. In the ICON, five occlusal trait scores are multiplied by their respective weights and computed in a final score. Then, the ICON score was further transformed to obtain two more different categories: (1) treatment need (categorized as: ‘no’, ICON ≤ 43; ‘yes’, ICON > 43); and (2) orthodontic complexity (easy, ICON < 29; mild, 29 ≤ ICON ≤ 50; moderate, 51 ≤ ICON ≤ 63; difficult, 64 ≤ ICON ≤ 77; very difficult, ICON > 77) [21].

OHRQoL was measured using the Portuguese validated version of the OHIP-14 questionnaire [23]. The OHIP-14 questionnaire assesses 14 items covering seven domains of oral health impact: functional limitation, physical pain, psychological discomfort, physical disability, psychological disability, social disability, and handicap [5]. Each item is rated on a five-point Likert scale coded as follows: 0—never; 1—hardly ever; 2—occasionally; 3—fairly often; and 4—very often. The OHIP-14 total score is then calculated as the sum of the 14 scores (from 0 to 56), with a higher score indicating more negative impacts and a lower OHRQoL. The OHIP-14 was also categorized as “frequently affected” vs. “less affected” OHRQoL following Kato et al.’s approach [24].

### 2.5. Measurement Reliability and Reproducibility

Measurement reproducibility was achieved, with one examiner (D.P.) trained and calibrated with another examiner considered the gold-standard (V.M.). Previous published orthodontic studies and more than five years of clinical experience were the criteria to be considered the gold-standard. Ten study casts from patients at the Department of Orthodontics (participants not included in the sample) were randomly selected and assessed. Then, both examiners employed the ICON to appraise inter-examiner reproducibility. Two weeks later, the same 10 dental casts were re-assessed by one examiner (D.P.) regarding intra-examiner reproducibility. The intra- and inter-examiner correlation coefficients were considered excellent for the ICON assessment (Cohen’s kappa = 1.00). 

### 2.6. Statistical Analysis

Analyses were performed as a function of sex and age range (0—“15–29 years old” or 1—“≥30 years old”).

Descriptive and inferential statistical methodologies were applied. All patients completed the questionnaires without missing data events. The OHIP-14 was calculated as a continuous measure and correspondent descriptive measures (mean and standard deviation [SD]) were computed. After the examination of data normality and homoscedasticity, the Mann–Whitney test was used to compare OHRQoL scores as a function of sex and age. For categorical variables, the analyses were performed using the chi-square test. Spearman’s rank correlation coefficient (rho) was used to analyze the correlation of OHIP-14 and ICON scores with the OHIP-14 total score, number of missing teeth, age and brushing frequency variables. The effect size of correlations was analyzed according to Cohen’s standard. Furthermore, a multiple forward stepwise linear regression analysis was carried out to evaluate the impact of those variables on the OHIP-14 total score. Next, a multivariable forward stepwise logistic regression was applied using the dichotomized dependent OHIP-14 variable “frequently affected” vs. “less affected”, as in [24]. The odds ratio (OR) and correspondent 95% confidence level intervals (95% CI) were calculated. Data were analyzed using IBM SPSS Statistics, v. 25, (IBM Corporation, Armonk, NY, USA). A level of significance of 5% was considered in all inferential analyses. 

## 3. Results

### 3.1. Sample Description

**Figure 1:**
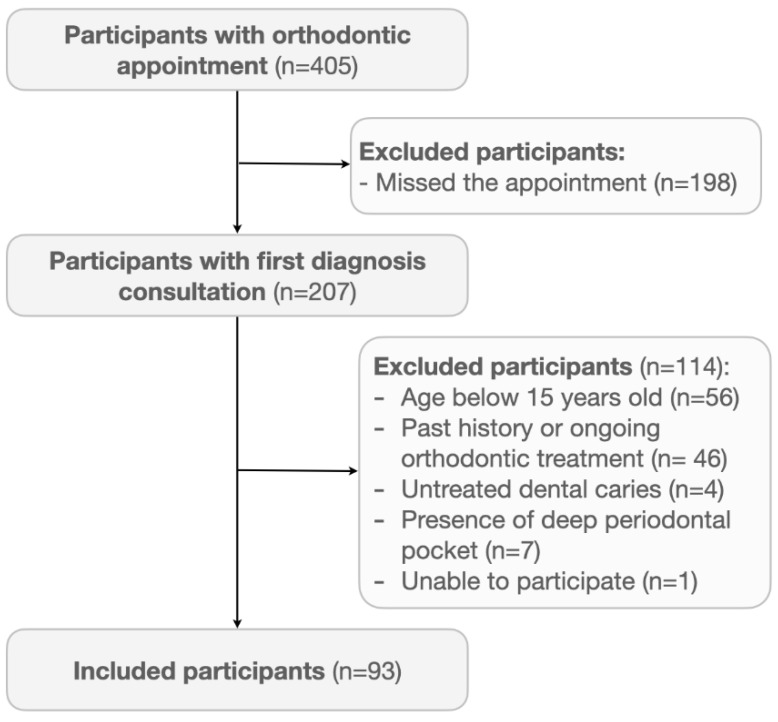
Participants flowchart.

From an initial sample of 405 patients with a scheduled orthodontic appointment, 207 presented to the Egas Moniz Dental Clinic Orthodontic Department. Of these, 93 participants (33 males and 60 females) met the eligibility criteria (Figure 1), aged 15 to 60 (Table 1). This sample had a majority of participants with a middle education level (58.1%), mainly with a single status (70.9%) and being students (47.3%) or being employed (47.3%). Regarding sex discrepancies, educational level (*p* = 0.022) and daily toothbrushing frequency (*p* = 0.019) presented differences. Participants aged 30 years old or older had significantly more missing teeth (*p* < 0.001), better self-reported interproximal hygiene (*p* < 0.001) and had different marital (*p* < 0.001) and occupation status (*p* < 0.001) than participants under 30 years old. Moreover, participants aged 30 years old or older had significantly less molars and premolars present (*p* < 0.001).

Furthermore, we compared the OHRQoL and orthodontic treatment need according to gender and age group (Table 2). On average, men reported significantly better OHRQoL (*p* = 0.005) and less functional limitation (*p* = 0.037), physical pain (*p* = 0.001), psychological discomfort (*p* = 0.016), physical disability (*p* = 0.001) and social disability (*p* = 0.036). Regarding orthodontic treatment needs and complexity, there were no differences between female and male groups. Nevertheless, participants under 30 years old had significantly less need for orthodontic treatment (*p* = 0.039). Furthermore, participants aged 30 years or older reported significantly worse OHRQoL (*p* = 0.042), as well as increased functional limitation (*p* = 0.017), physical disability (*p* = 0.039) and social disability (*p* = 0.016).

### 3.2. OHIP-14 and Covariates Impact

Then, we analyzed the strength and direction of association of OHIP-14 and ICON with other variables (Table 3). Age had a positive low correlation with OHIP-14 (ρ = 0.259, *p* < 0.05), and the number of missing teeth had a positive moderate correlation with OHIP-14 (ρ = 0.369, *p* < 0.001). Conversely, OHIP-14, the number of missing teeth, age and toothbrushing frequency had no significant correlation with ICON (*p* > 0.05).

To investigate which variables impacted the OHIP-14 total score, we conducted multiple linear regression analysis for the continuous measure of missing teeth (Table 4) and multiple logistic regression for the categorical presence of missing teeth (Table 5). Afterwards, the continuous measure of missing teeth was associated with OHIP-14, even adjusted for age, ICON and toothbrushing frequency (B = 1.48, SE = 0.57, *p* < 0.05). Notably, having missing teeth presents more risk towards worse OHIP-14 both in the crude model and adjusted models for sex, education, ICON need for treatment, job status and >30 years of age (B = 1.38, SE = 0.65, *p* < 0.05).

To investigate the association between ICON and OHIP-14 total score, we conducted a multiple logistic regression (Table 6). Remarkably, ICON presented no significant relationship, even when adjusted for multiple confounding variables (*p* > 0.05).

## 4. Discussion

As far as we are aware, this study may be the first unveiling the possible association of missing teeth with OHRQoL in patients seeking orthodontic care. Firstly, malocclusion treatment needs measured via ICON did not show an association with OHRQoL. Secondly, missing teeth, either as a continuous or categorical variable, was the most impactful confounding variable towards OHRQoL. The results also presented age as a significant variable in this equation, suggesting to further consider this construct in OHRQoL research in orthodontic patients.

We compared patients in two groups of age for several motives, and being 30 years old or older emerged as a likely important confounding variable, proposing this new hypothesis to future studies. We included participants aged 15 years old or older because late adolescence stands for a biological, psychological and social state of development, better established with adult cognitive skills well developed [25,26]. Additionally, a systematic review studying the impact of malocclusion and orthodontic treatment on OHRQoL also accounted for individuals with this age range [19]. Moreover, transitioning to adulthood has been prolonged in developed countries, as measured by the timing of traditional markers such as entrance to stable work, marriage, and parenthood [27], ranging from 18 to 29 years old [17]. Our findings corroborate that patients 30 years old or older seeking orthodontic treatment had lower perceived ORHQoL. Despite age having been previously reported to be associated with OHRQoL [28,29,30], these studies compared different age categories, and only one study had a population seeking orthodontic treatment, notwithstanding enrolling children and adolescents [30].

Oral conditions and sociodemographic variables may impact OHRQoL [29,30]. Several national surveys on OHRQoL carried out in several western European countries, Australia, and the USA also show that dental conditions contribute to lower quality of life [28,31,32,33,34,35,36,37]. Among different oral conditions, tooth loss represents the worst dental issue regarding oral health [38]. Additionally, the aggravation of tooth loss has been linked to diminished quality of life [39]. The worldwide prevalence of severe tooth loss declined between 1990 and 2010, from 4.4% to 2.4% [40]. Our data show that this population reported similar oral hygiene habits in agreement with other regional and national studies in Portugal [41,42,43], which may explain the significant number of missing teeth (Table S2). Notwithstanding, the loss of at least one tooth in this population (Table S2) was less prevalent than nationally reported [42], and the average missing teeth was lower than in the Lisbon metropolitan area study [43], considering the same age groups. Additionally, the prevalence of missing teeth was age-dependent, similar to these studies [41,42,43], and had the strongest association with OHRQoL. For this reason, evidence points to the importance of missing teeth in patients seeking orthodontic care, as they will be more vulnerable regarding OHRQoL.

The impact of malocclusion on OHRQoL is well documented [3,6,9,10,14]. However, a link between malocclusion with OHRQoL is not supported by our results. Our findings might be explained due to the age range (15 to 60 years old), considering age is a relevant clinical variable in orthodontic patients. In other words, previous studies focused on a particular age group and shed light on the association between OHRQoL and orthodontic treatment need [44,45,46]. Additionally, we focused on participants seeking orthodontic treatment, who may have neglected their oral health, introducing potential variability. Nevertheless, the association observed between OHRQoL and the number of missing teeth might be explained by a better self-perception of tooth loss compared to malocclusion.

### Strenghts and Limitations

The present study had some limitations. The cross-sectional study design applied in this study precludes cause and effect relationship inferences. Nevertheless, it comprises an exploratory analysis aimed at analyzing the complex relationship between several contributing factors for OHRQoL. The size of the sample is a shortcoming; therefore, the results should be interpreted with prudence, as this limits the validity of these results and warrants future confirmation with larger sample sizes. Still, this study comprised a 15-month inclusion period, followed a rigorous and up-to-date guideline and had a consecutive design, which may introduce some value to our results. Moreover, we applied widely accepted tools to measuring OHRQoL (OHIP-14) and orthodontic care needs (ICON).

## 5. Conclusions

Malocclusion does not have an impact on OHRQoL in this study population. Age and the number of missing teeth are significant variables on self-perceived OHRQoL in orthodontic patients. Likewise, the significance of the number of missing teeth was maintained even when analyzed simultaneously with age, ICON and toothbrushing OHRQoL impact on quality of life.

## Figures and Tables

**Table 1:** Sociodemographic characteristics, oral hygiene variables and number of missing teeth, according to sex, age and for overall participants (*N* = 93).

Variable	Female (*n* = 60)	Male (*n* = 33)	*p*-Value *	Age ≥15 and <30 (*n* = 57)	Age ≥30 (*n* = 36)	*p*-Value *	Total (*n* = 93)
Age, mean (SD)	31.6 (14.5)	27.1 (14.0)	0.078	19.7 (3.8)	46.2 (9.0)	**<0.001**	30.0 (14.4)
Education level, *n* (%)							
Middle	34 (56.7)	20 (60.6)	**0.022**	31 (54.4)	23 (63.9)	0.490	54 (58.1)
Higher	26 (43.3)	13 (39.4)		26 (45.6)	13 (36.1)		39 (41.9)
Marital Status, *n* (%)							
Single	40 (66.7)	26 (78.8)	0.176	55 (96.5)	11 (30.6)	**<0.001**	66 (70.9)
Married/Union of fact	17 (28.3)	4 (12.1)		2 (3.5)	19 (52.7)		21 (22.6)
Divorced	3 (5.0)	3 (9.1)		0	6 (16.7)		6 (6.5)
Occupation, *n* (%)							
Student	25 (41.7)	19 (57.6)	0.290	44 (77.2)	0	**<0.001**	44 (47.3)
Employed	32 (53.3)	12 (36.4)		10 (17.5)	34 (94.4)		44 (47.3)
Unemployed	3 (5.0)	2 (6.0)		3 (5.3)	2 (5.6)		5 (5.4)
TB frequency per day, *n* (%)							
3	10 (16.7)	8 (24.2)	**0.019**	7 (12.2)	11 (30.6)	0.064	18 (19.3)
2	49 (81.7)	20 (60.6)		47 (82.5)	22 (61.1)		69 (74.2)
1	1 (1.6)	5 (15.2)		3 (5.3)	3 (8.3)		6 (6.5)
Interproximal cleaning, *n* (%)							
Yes	16 (26.7)	6 (18.2)	0.505	5 (8.8)	17 (47.2)	**<0.001**	22 (23.7)
Powered toothbrush, *n* (%)							
Yes	6 (10)	6 (18.2)	0.422	6 (10.5)	6 (16.7)	0.587	12.0 (12.9)
Missing teeth, mean (SD)	2.3 (3.1)	1.2 (2.3)	0.118	0.4(1.0)	4.3 (3.2)	**<0.001**	1.9(2.9)
Type of tooth lost, *n* (%)							
Anterior teeth	6 (0.4)	3 (0.3)	0.917	2 (0.1)	7 (0.7)	0.138	9 (0.3)
Premolars	37 (2.2)	11 (1.2)	0.285	5 (0.3)	43 (4.3)	**<0.001**	48 (1.8)
Molars	92 (5.5)	27 (2.9)	0.066	15 (0.9)	104 (10.3)	**<0.001**	119 (4.6)

TB—Tooth brushing; * Mann–Whitney for continuous variables, chi-square test for categorical variables, *p* < 0.05 denoted in bold.

**Table 2:** OHIP-14 and ICON, according to sex and age and for overall participants (*N* = 93).

Variable	Female (*n* = 60)	Male (*n* = 33)	*p*-Value *	Age ≥15 and <30 (*n* = 57)	Age ≥30 (*n* = 36)	*p*-Value *	Total (*n*= 93)
OHIP-14 Total, mean (SD)	16.4 (11.8)	9.8 (8.8)	**0.005**	12.0 (10.1)	17.2 (12.4)	0.042	14.1 (11.3)
OHIP-14 domains, mean (SD)Functional limitation	0.7 (1.2)	0.3 (0.8)	**0.037**	0.4 (0.8)	0.9 (1.32)	**0.017**	0.6(1.1)
Physical pain	1.8 (1.3)	0.9 (1.0)	**0.001**	1.3 (1.2)	1.77 (1.35)	0.063	1.5 (1.3)
Psychological discomfort	1.99 (1.4)	1.32 (1.4)	**0.016**	1.6 (1.4)	2.05 (1.44)	0.060	1.8 (1.5)
Physical disability	1.03 (1.3)	0.32 (0.6)	**0.001**	0.6 (1.0)	1.06 (1.27)	**0.039**	0.8 (1.1)
Psychological disability	1.5 (1.4)	1.1 (1.3)	0.064	1.2 (1.3)	1.6 (1.37)	0.055	1.3 (1.4)
Social disability	0.6 (1.0)	0.3 (0.9)	**0.036**	0.4 (1.0)	0.65 (1.0)	**0.016**	0.5 (1.0)
Handicap	0.7 (1.1)	0.6 (0.93)	0.937	0.7 (1.1)	0.6 (1.0)	0.768	0.6 (1.0)
ICON treatment need, *n* (%)							
No	24 (40.0)	12 (36.4)	0.800	27 (47.4)	9 (25.0)	**0.039**	36 (38.7)
Yes	36 (60.0)	21 (63.6)		30 (52.6)	27 (75.0)		57 (61.3)
ICON complexity, *n* (%)							
Easy	5 (8.3)	4 (12.1)	1.000	9 (15.8)	0	0.744	9 (9.7)
Mild	32 (53.4)	14 (42.4)		26 (45.6)	20 (55.5)		46 (49.5)
Moderate	10 (16.6)	3 (9.1)		7 (12.3)	6 (16.7)		13 (13.9)
Difficult	5 (8.3)	7 (21.2)		6 (10.5)	6 (16.7)		12 (13)
Very difficult	8 (13.4)	5 (15.2)		9 (15.8)	4 (11.1)		13 (13.9)

OHIP-14—Oral Health Impact Profile-14; index of complexity, outcome and need—ICON; standard deviation—SD; * Mann–Whitney for continuous variables, chi-square test for categorical variables, *p* < 0.05 denoted in bold.

**Table 3:** Correlation between ICON and OHIP-14 total score, number of missing teeth, age and toothbrushing frequency (*n* = 93).

Variable	ICON	OHIP-14
ICON	-	0.194
OHIP-14	0.194	-
Number of missing teeth	0.159	0.369 **
Age	0.124	0.259 *
Tooth brushing frequency	−0.186	−0.153

Index of complexity, outcome and need—ICON; OHIP-14—Oral Health Impact Profile-14; Pearson ρ correlation, * *p* < 0.05, ** *p* < 0.001.

**Table 4:** Crude and adjusted linear regression models and correspondent standard error of OHIP-14 total score towards number of missing teeth (*n* = 93).

	Number of Missing Teeth
Model 1	1.46 (0.39) ***
Model 2	1.54 (0.57) **
Model 3	1.45 (0.57) *
Model 4	1.48 (0.57) *

Model 1—unadjusted model; model 2—includes adjustment for age; model 3—includes adjustment for age and ICON; model 4—includes adjustment for age, ICON and toothbrushing frequency. * *p* < 0.05; ** *p* < 0.01; *** *p* < 0.001. OHIP-14—Oral Health Impact Profile-14; index of complexity, outcome and need—ICON.

**Table 5:** Crude and adjusted logistic regression models and correspondent standard error of frequently affected OHIP-14 total score towards the presence of missing teeth (*n* = 93).

	Presence of Missing Teeth
Model 1	1.20 (0.46) **
Model 2	1.14 (0.46) *
Model 3	1.15 (0.47) *
Model 4	1.07 (0.48) *
Model 5	1.13 (0.54) *
Model 6	1.38 (0.65) *

Model 1—unadjusted model; model 2—includes adjustment for sex; model 3—includes adjustment for sex and education; model 4—includes adjustment for sex, education and ICON need for treatment; model 5—includes adjustment for sex, education, ICON need for treatment and job status; model 6—includes adjustment for sex, education, ICON need for treatment, job status and ≥30 years of age. * *p* < 0.05; ** *p* < 0.01. OHIP-14—Oral Health Impact Profile-14; index of complexity, outcome and need—ICON.

**Table 6:** Crude and adjusted logistic regression models and correspondent standard error of frequently affected OHIP-14 total score towards the presence of missing teeth (*n* = 93).

	ICON Need for Treatment
Model 1	0.55 (0.44)
Model 2	0.62 (0.45)
Model 3	0.63 (0.46)
Model 4	0.36 (0.49)
Model 5	0.35 (0.49)
Model 6	0.37 (0.49)

Model 1—unadjusted model; model 2—includes adjustment for sex; model 3—includes adjustment for sex and education; model 4—includes adjustment for sex, education, and presence of missing teeth; model 5—includes adjustment for sex, education, presence of missing teeth and job status; model 6—includes adjustment for sex, education, presence of missing teeth, job status and ≥30 years of age. OHIP-14—Oral Health Impact Profile-14.

## Data Availability

Data may be available upon reasonable request.

## Supplementary Materials

The following are available online at https://www.mdpi.com/article/10.3390/ijerph18137145/s1: Table S1: STROBE Statement.; Table S2: Number of participants per missing teeth, according to sex and age and for overall participants (*N* = 93).
